# Leaf mycobiome and the success of *Hymenoscyphus fraxineus* in completing its life cycle depend on the canopy position of common ash

**DOI:** 10.3389/fmicb.2025.1696858

**Published:** 2025-12-09

**Authors:** Feng Long, Chatchai Kosawang, Lene R. Nielsen, Ari M. Hietala

**Affiliations:** 1 Department of Geosciences and Natural Resource Management, University of Copenhagen, Frederiksberg, Denmark; 2 Norwegian Institute of Bioeconomy Research, Steinkjer, Norway

**Keywords:** saplings, mature trees, *Hymenoscyphus fraxineus*, fungal community, diversity

## Abstract

**Introduction:**

Common ash (*Fraxinus excelsior*) is threatened by an invasive Asian-origin ash dieback pathogen, *Hymenoscyphus fraxineus*. The pathogen establishes leaf infection with the aid of wind-borne ascospores, followed by mycelial spread from the leaf into the shoot before autumn senescence. Whether the mycobiome in living leaves and the fructification success of this fungus in overwintered leaves differ between saplings and different crown layers of large trees is poorly understood.

**Methods:**

We pursued these questions by ITS1-based amplicon sequencing and recording of the number of pathogen ascocarps formed on leaf debris.

**Results:**

Fungal diversity (Shannon and Simpson indices) and richness (Chao1) in leaves differed significantly between saplings and the upper or lower light canopy of large trees. Saplings showed the most diverse fungal communities, and the upper light canopy had the least diverse ones. In saplings, the two most dominant fungi were an unknown ascomycete and *H. fraxineus* (combined read proportion 27.2%), while this unknown ascomycete and genus *Aureobasidium* were the most common fungi in upper (combined read proportion 70.2%) and lower (combined read proportion 52.0%) light canopy of large trees. The number of *H. fraxineus*-like ascocarps produced on petiole/rachis tissues after overwintering was, on average, 7.5 for saplings and 1.4 for large trees (canopy layers combined); saplings differed significantly from most of the large trees in this respect. Leaves from large trees with no defoliation also produced *H. fraxineus*-like ascocarps.

**Discussion:**

The potential interactions between *H. fraxineus* and phyllosphere fungal community and the implications of our findings to management of ash stands are discussed.

## 1 Introduction

The invasive ascomycete *Hymenoscyphus fraxineus* (syn. *H. pseudoalbidus*, anamorph *Chalara fraxinea*) (Kowalski, 2006; Queloz et al., 2011; Baral and Bemmann, 2014) has been threatening common ash (*Fraxinus excelsior* L.) across Europe for the past decades. In its presumed native range in Asia, the fungus appears to be primarily a leaf endophyte on local *Fraxinus* species, as no crown dieback of ash has been observed in forests where this fungus occurs (Zhao et al., 2012; Zheng and Zhuang, 2014; Inoue et al., 2019). In addition to leaf infection, the European common ash often develops *H. fraxineus*-associated root-collar lesions, with frequent secondary colonization by *Armillaria* spp. (Chandelier et al., 2016; Marçais et al., 2016; Enderle et al., 2017b). According to the current common ash-*H. fraxineus* disease model, the pathogen spreads by ascospores, which are produced by fruiting bodies that form on shed ash leaves after overwintering. Deposition of the wind-disseminated ascospores on ash leaf surface is followed by ascospore germination, leaf penetration by the germ tube and mycelial spread through the leaf petiole base into shoots and twigs before leaf shed (Haňáčková et al., 2017). In addition, shoot lenticels (Nemesio-Gorriz et al., 2019) and direct penetration of epidermis of young shoots (Mansfield et al., 2019) may serve as entrance points for pathogen ascospore germlings to stem tissues.

At an epidemic level of ash dieback, one square meter of ash forest floor can have several thousand fruiting bodies of *H. fraxineus* at the time of sporulation (Hietala et al., 2013). This causes a tremendous local propagule pressure that facilitates also the long-distance spread of *H. fraxineus*, which is typical of invasive species. On common ash, the disease is manifested by necroses in shoots and twigs, resulting in gradual crown decline and eventual tree death (Przybył, 2002; Kowalski et al., 2017). Mortality rates of up to ∼80% have been recorded in common ash stands (Coker et al., 2019). However, most published mortality estimates derive from surveys of mature trees while observations of seedlings and saplings are under-represented, as they fall outside routine inventory thresholds and are harder to monitor (but see Coker et al., 2019). Because many species are strongly or obligately associated with common ash, the disease poses cascading impacts upon the biodiversity associated with this keystone tree species (Mitchell et al., 2014).

The invasiveness of *H. fraxineus* in Europe is enabled by its efficient capture of the leaf vein system of common ash, its sporulation niche. Several studies have been conducted to profile the leaf mycobiome of common ash to understand the fungal community dynamics during the growing season and the association of *H. fraxineus* with indigenous fungi inhabiting the tissue (Cross et al., 2017; Griffiths et al., 2019; Agostinelli et al., 2021). The data show that already in June, when the sporulation of *H. fraxineus* typically initiates in Norway (Hietala et al., 2013), ash leaf tissues are colonized by a wide range of filamentous fungi and yeasts with epiphytic, endophytic, biotrophic, necrotrophic and/or saprotrophic feeding modes (Cross et al., 2017). Ash dieback affects common ash trees of all ages, although younger trees are usually killed faster than larger trees (Timmermann et al., 2020; Madsen et al., 2021). The prior studies describing ash leaf mycobiome and its seasonal dynamics have focused on saplings or smaller trees (Cross et al., 2017; Griffiths et al., 2019; Agostinelli et al., 2021), perhaps because of their rapid dieback but also because of the technical challenges in sampling leaves from large trees. Prior to the arrival of ash dieback in Germany, Reiher and colleagues (Reiher, 2011; Scholtysik et al., 2012) considered whether the leaf mycobiome of common ash differs between the light and shade crown of dominant large trees and the crown of understory saplings. In their studies, the ash leaflet samples were collected across summer and autumn with the aid of a crane and subjected to surface sterilization followed by ITS-based taxonomic characterization of the cultured fungi. These studies revealed spatiotemporal niche partitioning in abundance and species composition of fungi that depended on the time of season and the canopy position. Across all canopy layers, isolation frequency and endophytic species richness increased over the growing season from May to October. Understory saplings and shade crown of large trees showed consistently higher isolation frequency of fungi, species richness, and diversity than the light crown of large trees. In the autumn, species of *Alternaria*, *Ramularia*, *Septoria*, *Phoma* and *Colletotrichum* were the most common fungi in the understory saplings, with rather similar isolation frequencies. In the light canopy of large trees, *Alternaria* spp. were by far the most commonly isolated species in the autumn (Reiher, 2011; Scholtysik et al., 2012).

The faster dying of younger ash trees in forests infested by *H. fraxineus* is likely related to the high propagule pressure at lower canopy levels in ash forests, which facilitates faster stem colonization and subsequent girdling. Chandelier et al. (2014) showed that in ash forests, the ascospore level in the air can be up to 100-fold lower already at a height of 3 m in comparison to a height of 0.5 m. Concerning management methods to reduce ash mortality, silvicultural measures such as the removal of trees that contribute most to life cycle completion by *H. fraxineus* could be beneficial. Up to 5 percent of common ash trees show increased tolerance to ash dieback in the sense that they show little or no shoot dieback in forests with epidemic levels of the disease. It is known that leaves of ash species with high tolerance to shoot dieback can support even abundant ascomata production by *H. fraxineus* (Nielsen et al., 2017), indicating that susceptibility to leaf infection and shoot dieback are governed by distinct traits. It has also been shown that saplings of common ash trees with no shoot symptoms can support the life cycle completion of this pathogen (Marçais et al., 2023). The extent to which the foliage in the light-exposed canopy of mature ash trees supports the completion of the pathogen lifecycle is poorly understood. To expand on the findings of prior studies and the research questions that have arisen from them, we carried out a metabarcoding study of the ash leaf mycobiome in a multi-storied natural forest of common ash to consider the following hypothesis: the canopy position of an ash tree affects not only the composition and diversity of leaf mycobiome but also the success of *H. fraxineus* in infection of leaf tissues and completion of its life cycle.

## 2 Materials and methods

### 2.1 Sample collection and preparation

Leaf samples were collected from nine mature ash trees (18–30 m) with different degrees of defoliation and up to eight understory ash saplings grown in the nature reserve forest in Hindrem in central Norway. Three to four twigs were collected from each mature tree by a DJI Matrice 300 RTK drone (DJI, Shenzhen, China) equipped with the DeLeaves^®^ canopy sampling tool (Outreach Robotics, Sherbrooke, Canada). Of these, two were from the uppermost part of the light canopy and one or two twigs from 3 to 4 m below the top of light canopy. For each twig, three compound leaves were randomly sampled, and one basal, one central and one apical leaflet from each compound leaf were combined, ground, and treated as one sample. In total, 35 samples were obtained (four samples per tree, except one tree had only three samples). In the understory of the mature trees, eight saplings were alive at the time of sampling, each with very few leaves. Therefore, the leaves from these saplings were pooled, and randomly divided into four samples meaning that comparisons at the individual level were not possible. Sampling was performed on the 19th of September 2022, and the samples were stored at -20°C until further processing. Tree phenotype, including tree height (m), diameter at breast height (DBH, cm), defoliation degree [three classes: low (0–10%), intermediate (10–30%), and high (30–80%)] and sampling height (m), was recorded at the time of sampling (Table 1). It is noteworthy that, besides *H. fraxineus*, the ash sawfly *Tomostethus nigritus* had also been present in the forest for 2–3 years by the time of sampling. Its larvae can cause substantial early-season, patchy defoliation with possible refoliation later; repeated outbreaks reduce tree vigor (Soldi et al., 2021; Meshkova et al., 2017). Thus, a portion of crown thinning likely reflects sawfly herbivory rather than ash dieback alone, so we treat canopy scores as a composite signal when we analyze the effect of defoliation on the community.

**TABLE 1:** The proportion (%) of petioles with *H. fraxineus* fruiting bodies (FBs) within the basal 5 cm of the petiole, and the number of such fruiting bodies per petiole/rachis tissue for the large trees and saplings after a 1-year-incubation period in the forest floor.

Tree ID	Height	DBH	Defoliation	Upper sampling	Lower sampling	Proportion of petioles	Number of FBs
	m	cm	%	Height, m	Height, m	With FBs at base (%)	Per petiole
Tree 1	18.0	35.0	10.0%	17.0–18.0	14.0–15.0	0.0 ± 0.0 b	0.3 ± 0.3 bc
Tree 2	20.0	45.0	10–20.0%	19.0–20.0	15.0–18.0	16.3 ± 18.8 b	2.4 ± 2.8 bc
Tree 3	27.0	47.0	0.0%	26.0–27.0	24.0–25.0	2.8 ± 3.2 b	1.9 ± 2.6 bc
Tree 4	28.0	45.0	0.0%	27.0–28.0	25.0–26.0	2.8 ± 5.5 b	2.7 ± 3.1 abc
Tree 5	28.0	60.0	30.0%	27.0–28.0	25.0–26.0	12.7 ± 21.9 b	1.0 ± 1.8 bc
Tree 6	28.0	58.0	50.0%	27.0–28.0	25.0–26.0	5.0 ± 5.8 b	0.9 ± 1.1 bc
Tree 7	30.0	75.0	70–80.0%	29.0–30.0	27.0–28.0	14.5 ± 20.7 b	2.6 ± 1.0 ab
Tree 8	24.0	47.0	30–40.0%	23.0–24.0	21.0–22.0	0.0 ± 0.0 b	0.3 ± 0.5 c
Tree 9	27.0	53.0	0.0%	26.0–27.0	24.0–25.0	0.0 ± 0.0 b	0.7 ± 0.8 bc
Saplings	2.0–5.0	< 5.0	not assessed	5.0	1.0	39.0 ± 4.0 a	7.5 ± 2.7 a

Mean and standard deviation values are shown in the table. The data for the lower and upper light canopy of a large tree were pooled together as there were no sampling position-specific patterns in the production of *H. fraxineus* fruiting bodies. Different lowercase letters indicate significant differences among groups (Kruskal-Wallis and Dunn’s *post hoc*, *P* < 0.05).

### 2.2 DNA extraction, PCR and ITS1 sequencing

DNA was extracted using Quick-DNA Plant/Seed Miniprep Kit (Zymo research, Irvine, CA, United States) following the manufacturer’s instructions. The concentration of the DNA was determined using Qubit 3.0 (Invitrogen, Carlsbad, CA, United States) with Qubit™ dsDNA High Sensitivity Assay Kits (Invitrogen, Oregon, United States). A two-step PCR approach was used to construct amplicon libraries for fungal community analysis. The Internal Transcribed Spacer 1 (ITS1) region of the nuclear ribosomal gene cluster was first amplified using the primer pair BITS and B58S3 (Bokulich and Mills, 2013) equipped with appropriate Illumina adapters. The PCR reactions were set up using 10 ng DNA template and Phusion High-Fidelity PCR Master Mix (Thermo Scientific, Waltham, MA, United States) on a T100 Thermal cycler (Bio-RAD, Hercules, CA, United States) as follows: 98°C for 30 s, followed by 32 cycles of 98°C for 10 s, 57°C for 30 s, 72°C for 15 s before final extension at 72°C for 5 min. Each sample was amplified in triplicate, which were later pooled and visualized on a 1% agarose gel. Dual barcodes from Nextera XT v2 (Illumina, San Diego, CA, United States) were introduced to each sample during the second PCR with the same cycling conditions, except that the annealing temperature was at 56°C and PCR cycles were limited to 12. The PCR products were purified using PureLink™ PCR Micro Kit (Thermo Fisher Scientific, Massachusetts, United States), quantified with Qubit, and pooled at an equimolar ratio before being cleaned up and size-selected by NucleoMag kit (MACHEREY-NAGEL, Düren, Germany). The ITS1 amplicon libraries were sequenced using Illumina MiSeq with v3 chemistry (2 × 300 bases) at Macrogen Europe (Amsterdam, The Netherlands).

### 2.3 Bioinformatic and statistical analysis

Forward reads were used in this study as recommended by Pauvert et al. (2019); this because the use of forward reads allows to capture also fungi with longer ITS sequences. *Hymenoscyphus fraxineus* has a long insert in the ITS1 region, which makes the amplification of its ITS1 region in fungal species rich environmental samples very inefficient (Cross et al., 2017). Low-quality bases (*q* < 20) and short reads ( < 140 bases) were removed using a BBduk tool of BBTools suite.^1^ The high-quality reads were parsed to QIIME2 version 2022.11 (Bolyen et al., 2019), where primers and adapters were removed using q2_cutadapt (Martin, 2011). Operational Taxonomic Units (OTUs) were assigned at 99% sequence similarity against the UNITE fungal ITS database version 9.0 (released 2023-7-18) (Abarenkov et al., 2023) using VSEARCH (Rognes et al., 2016) and the close-reference OTU approach of QIIME2. Low-abundance OTUs were removed using 0.005% as a cut-off as recommended by Bokulich and Mills (2013).

After quality control, primer-trimming and adapter removal, we retained 68.2% of raw reads (914,757 of 1,341,400). The high-quality reads ranged from 14,403 to 38,520 across 39 samples. A total of 191 fungal OTUs were identified after taxonomic assignment from leaf samples and the specific details for each OTU are shown in Supplementary Table 1. All samples were rarefied to 14,403 reads per sample. Alpha (Observed OTU, Chao1, Shannon and Simpson) and beta (Bray-Curtis) diversity indices and principal component analysis were computed in R version 4.2.2 (R Core Team, 2022) using the following packages*: phyloseq v. 1.42.0* (McMurdie and Holmes, 2013) and *Vegan v. 2.6-4* (Dixon, 2003). The UpSet plot was illustrated using *ComplexUpset v.1.3.6* (Lex et al., 2014; Krassowski, 2020). Pairwise Kruskal-Wallis tests were applied to test for significant differences in alpha diversity of the mycobiome associated with different defoliation degrees and sampling positions. We also calculated the permutational multivariate analysis of variance (PERMANOVA, permutations = 999) to consider differences in the composition of mycobiome between trees and saplings.

To further characterize potential fungal interactions of phyllosphere mycobiome communities, we performed network analyses of the fungal communities associated with the mature trees (the data from the upper and lower light canopy were pooled together) and saplings. The co-occurrence networks were analyzed individually using all OTUs. Robust correlations with Spearman correlation coefficients (*P* > 0.6) and false discovery rate-corrected *p* < 0.05 were used to construct networks using the *psych package v. 2.3.9* of R (Revelle, 2017). The topological characteristics of networks (average degree, modularity, graph density, average clustering coefficient, and number of modules) were calculated and visualized in *Gephi v. 0.10* software (Bastian et al., 2009). Unless otherwise stated, the analyses were performed in R v. 4.2.2 and the above-described R packages.

### 2.4 qPCR estimation of the total fungal DNA level and that of *H. fraxineus* in the leaflets subjected to metabarcoding

The forward primer FungiQuant-F (5′-GGRAAACTCACCAGGTCCAG-3′), the reverse primer FungiQuant-R (5′-GSWCTATCCCCAKCACGA-3′) and the probe FungiQuant-Prb (5′-FAM-TGGTGCATGGCCGTT-BHQ1-3′ (Liu et al., 2012) were used for quantification of total fungal DNA. For detection of *H. fraxineus*, the forward primer Cfrax-F (5′-ATTATATTGTTGCTTTAGCAGGTC-3), the reverse primer Cfrax-R (5′-TCCTCTAGCAGGCACAGTC-3′) and the probe Cfrax-P (5′-FAM-CTCTGGGCGTCGGCCTCG- BHQ1-3′ (Ioos et al., 2009) were used. The primer and probe concentrations used for both assays were 300 and 100 nM, respectively. PCR reactions were set up with SSoAdvanced Universal Probes Supermix (Bio-Rad, CA, United States) according to manufacturer instructions on a CFX Connect™ Real-Time System (Bio-Rad, CA, United States). PCR cycling parameters were 95°C for 10 min, followed by 40 cycles of 95°C for either 3 s (*H. fraxineus*) or 15 s (FungiQuant) and 65°C for 25 s (*H. fraxineus*) or 55 s (FungiQuant). Log-dilution series were prepared for all the experimental samples; 10- and 100-fold diluted samples were used as templates for *H. fraxineus* qPCR and 10-, 100-, and 1,000-fold dilutions were used as templates for FungiQuant qPCR. Three microliters of the DNA solution were used as the template for each 20-μL reaction, and each biological replicate was repeated twice. The CT (cycle threshold) values (lower Ct = higher DNA abundance) were recorded at a standardized fluorescence level within the exponential phase of the *H. fraxineus* and FungiQuant qPCR assays. When the delta CT value between consequent template dilutions was close to 3.3, the template dilutions were considered free of inhibitory compounds and represent the linear range of detection of the target sequence. Ten-fold diluted samples were concluded to provide reliable CT values in the *H. fraxineus* qPCR (mean delta CT between 10- and 100-fold dilutions 3.18), while 1,000-fold diluted samples were concluded to provide reliable CT values in the FungiQuant qPCR (mean delta CT between 100- and 1,000-fold dilutions 3.25).

### 2.5 Formation of *H. fraxineus* fruiting bodies in petioles from saplings and large trees

To assess the production of *H. fraxineus* ascocarps in petioles from large trees and saplings, leaflets were removed from randomly selected compound leaves. The petiole/rachis tissues were packed in 1-mm-mesh nylon bags that were labeled with sample ID. Each nylon bag contained typically 15–30 petioles. Two nylon-bag replicates were prepared for the upper light canopy and two for the lower light canopy of each large tree. The petioles from saplings were pooled together owing to a low number of leaves present in each sapling and randomly divided into three nylon bags. The nylon bags were anchored with sticks to the ground at Hindrem Nature Reserve and covered with leaf debris on 30 September 2022. The experiment was harvested on 9 August 2023, at a time when there was a large amount of *H. fraxineus*-like actively sporulating ascomata at the stand (touching of petioles with ascomata triggered the release of a spore cloud). Identification of putative *H. fraxineus* ascomata was based on macroscopic features (Baral and Bemmann, 2014). The number of *H. fraxineus* ascomata per petiole/rachis was counted as was the proportion of petioles hosting *H. fraxineus* ascomata within 5 cm from petiole base. Because the data deviated from normal distribution, the significance of differences in *H. fraxineus* ascomata production between large trees (data from the lower and upper light canopy of a tree were pooled together) and saplings was considered with the Kruskal-Wallis test, followed by Dunn’s *post-hoc* test. *P*-values below 0.05 were considered significant.

## 3 Results

### 3.1 Phyllosphere fungal community composition and diversity of *Fraxinus excelsior*

In total, we identified 191 fungal OTUs. After taxonomic assignment, the represented fungi included 86 OTUs in the phylum *Ascomycota*, 87 OTUs in the phylum *Basidiomycota*, and 18 unknown fungi. *Ascomycota* formed 85.1 and 62.3 % and *Basidiomycota* 11.5 and 33.5% of the sequence reads in large trees and saplings, respectively. Unidentified Ascomycetes, and OTUs in the classes *Dothideomycetes*, *Sordariomycetes*, *Leotiomycetes* and *Taphrinomycetes* were the most common *Ascomycota*, with respective sequence read proportions of 39.6, 34.4, 5.3, 2.9, and 2.8% in large trees and 16.6, 15.2, 26.9, 1.1, and 2.4% in saplings (Supplementary Figure 1A). For *Basidiomycota*, the most common OTUs represented the classes *Agaricomycetes*, *Tremellomycetes* and *Pucciniomycetes*, accounted for 3.2, 5.2, and 2.7% of reads in large trees and 16.2, 15.9, and 0.9% in saplings, respectively (Supplementary Figure 1A).

**FIGURE 1:**
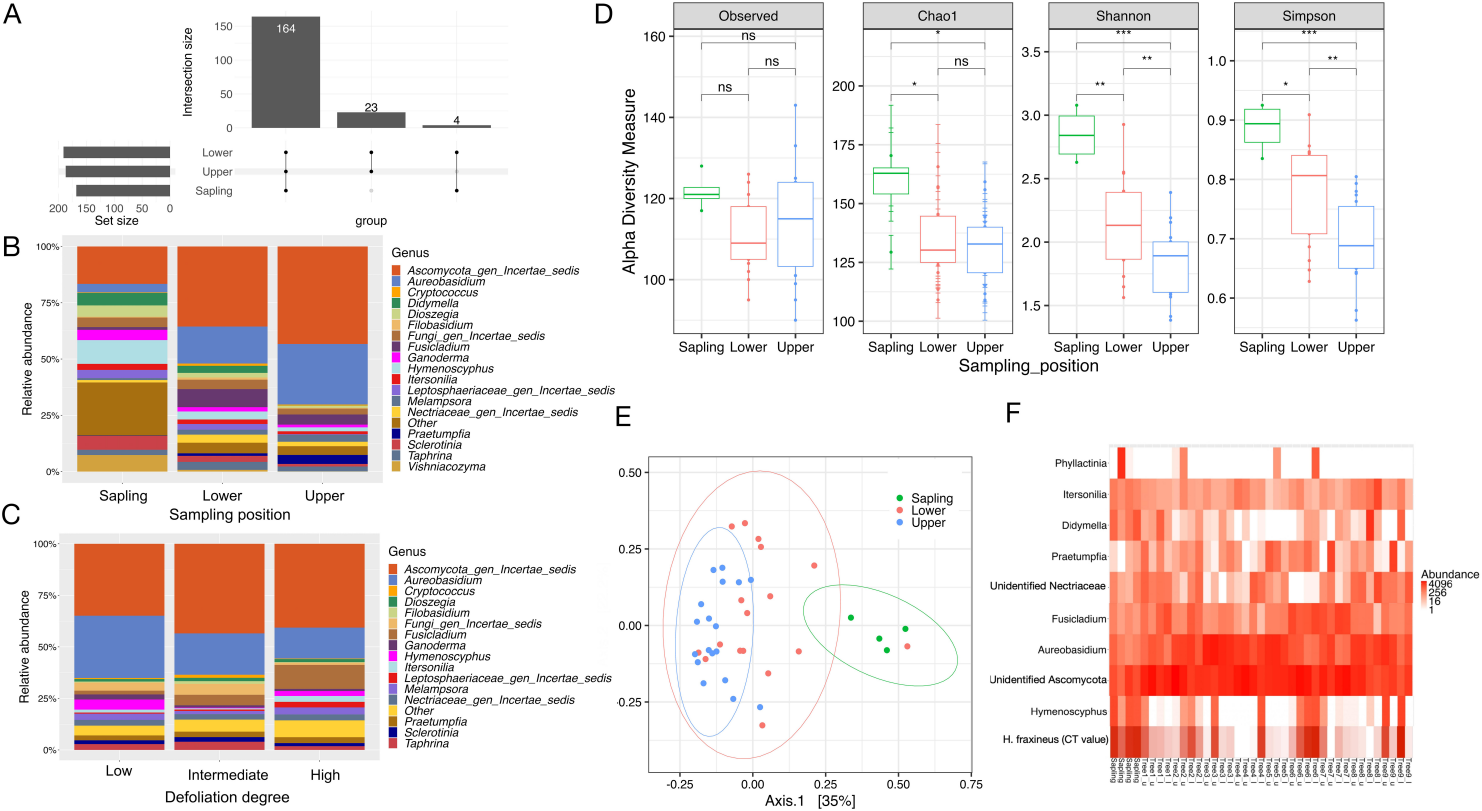
(A) UpSet plot showing the number of fungal taxa detected by metabarcoding that are among or specific to each canopy. (B) Relative abundance of the most common fungal genera (at least 15% prevalence in at least one sample) according to sampling positions and (C) defoliation degree of mature trees. (D) Fungal community diversity, including richness (observed OTUs, Chao 1), and diversity (Shannon and Simpson indices) (*denotes significance: *P < 0.05, **P < 0.01, ***P < 0.001). (E) Principal coordinates analysis (PCoA) of phyllosphere mycobiome associated with saplings and upper and lower crown of mature ash trees. Ellipses represent 95% confidence intervals for each sample type. (F) Heatmap showing OTUs that represent at least 15% of reads in at least one sample. For reference purposes, CT values of *H. fraxineus*, measured by the pathogen-specific qPCR, are expressed as a heatmap in the last row, the lower the CT value, the darker the color.

Of the 191 fungal OTUs, 164 were shared among saplings and both the upper and lower crown layers of the mature trees, while 23 OTUs were unique to the mature trees (Figure 1A). The two most common OTUs in saplings were an unknown *Ascomycete* and *Hymenoscyphus fraxineus*, which accounted for 16.6 and 10.5% of total sapling reads, respectively. The same unknown ascomycete and *Aureobasidium pullulans* (de Bary) G. Arnaud (1918) dominated large trees, representing 39.6 and 21.7% of total reads in mature trees, respectively. *H. fraxineus* had a sequence read proportion of only 2.6 % in mature trees (Figure 1B). No unique OTUs were found in saplings, while the 23 unique OTUs discovered in mature trees (Supplementary Table 1) were assigned to 14 genera. These included species in the genera *Blumeria*, *Sporobolomyces*, *Cortinarius*, *Gymnopus*, *Lophodermium*, and *Melanohalea*, and together account for only 0.24% of total reads across all samples.

The relative abundance of some genera could have reflected the sampling position. For instance, the relative abundance of *Hymenoscyphus*, *Didymella, Sclerotinia*, and *Dioszegia* showed a trend of decrease from sapling to upper light canopy, while *Aureobasidium* and the unknown ascomycete showed an opposite tendency (Figure 1B). Concerning the relation between mycobiome composition and defoliation degree in large trees, there was no obvious pattern except that the relative abundance of *Aureobasidium* showed a trend of decrease along with an increase in the defoliation degree (Figure 1C). We found that the leaf mycobiome differed among different sampling positions in terms of alpha diversity indices (Chao1, Shannon, and Simpson) (Figure 1D). Specifically, the communities associated with saplings had higher Chao 1, Shannon and Simpson indices than those of the lower (Kruskal-Wallis, *P* < 0.05, *P* < 0.01, and *P* < 0.05, respectively, *N* = 39) and upper canopies (Kruskal-Wallis, *P* < 0.05, *P* < 0.001, and *P* < 0.001, respectively, *N* = 39) of mature trees (Figure 1D). However, we found no significant difference in alpha diversity among defoliation degrees (Supplementary Figure 1D).

With the exception of one sample from lower canopy of large trees, Principal Coordinate Analysis (PCoA) separated samples from saplings and large trees in two clusters. There was more overlap between samples from lower and upper light canopy layers (Figure 1E). PERMANOVA analysis showed that the differences in fungal community composition between saplings and the lower or upper crown of large trees and between the lower and upper crown of large trees were statistically significant (*P* = 0.001, *R*^2^ = 0.213; *P* = 0.001, *R*^2^ = 0.431; *P* = 0.01, *R*^2^ = 0.086, respectively, *N* = 39). Significant differences in fungal community composition were also observed between trees with different degrees of defoliation (*P* = 0.009, *R*^2^ = 0.134, *N* = 35) (Supplementary Table 2). However, no obvious differences were observed based on PCoA (Supplementary Figure 1E).

### 3.2 Abundance of *Hymenoscyphus fraxineus* in ash leaves increases along with fungal diversity irrespective of sampling position

**FIGURE 2:**
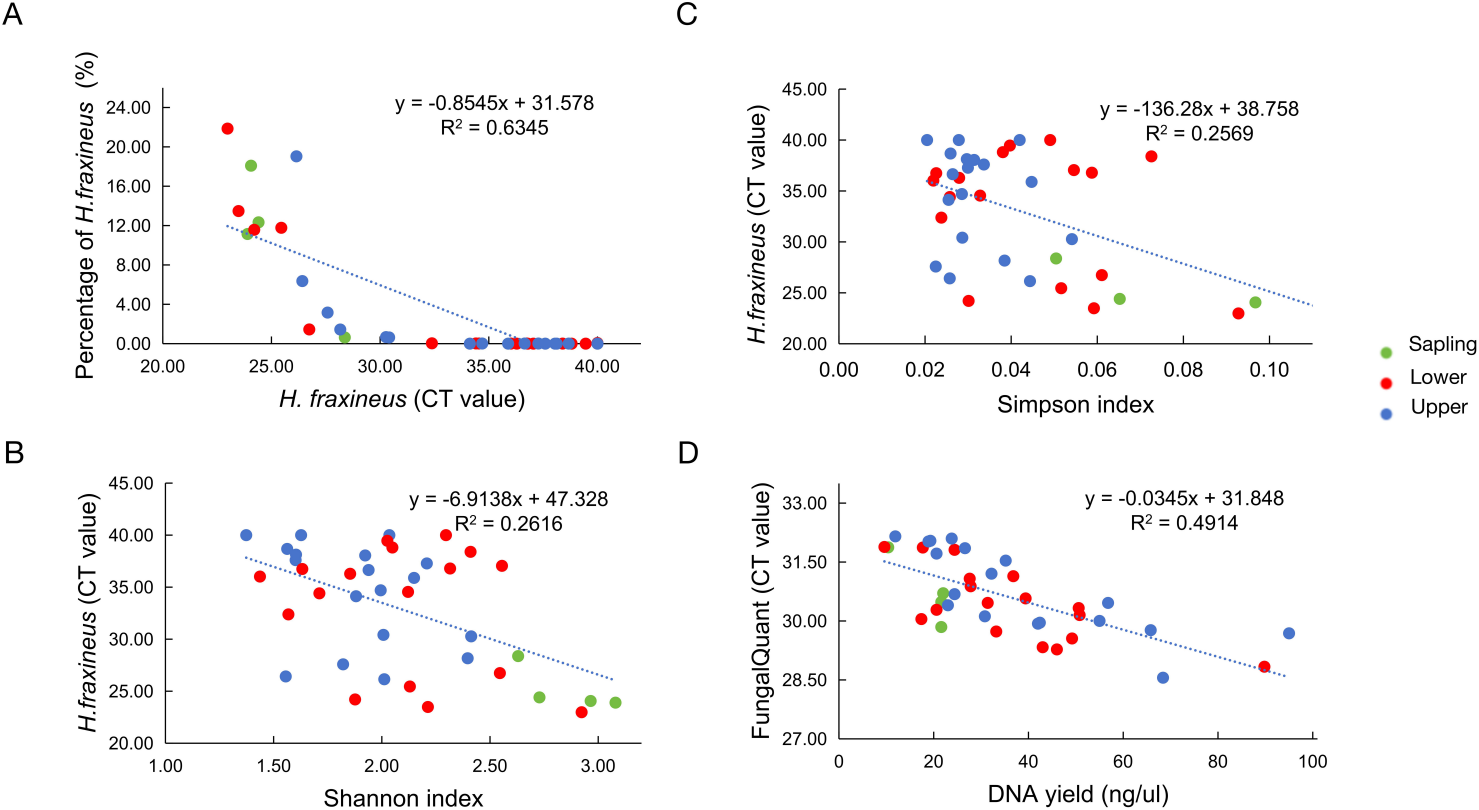
Linear relationships between (A) percentage of *H. fraxineus* sequence reads and its qPCR-derived CT value. (B,C) CT value of *H. fraxineus* and fungal community diversity (Shannon and Simpson indices). (D) Total DNA yield (ng/μL) and CT values from FungalQuant. Lower CT values indicate higher DNA abundance. The dot colors represent sampling position, while the trendlines illustrate overall trends with linear regression.

The sequence read proportions of *H. fraxineus* covaried (*R*^2^ = 0.6345, linear regression, y = -0.8545 + 31.578) with qPCR-derived CT values for the fungal community (Figures 1F, 2A). The proportion of *H. fraxineus* reads was higher in saplings than in large mature trees but the difference was significant only in comparison to the upper canopy. The CT value of *H. fraxineus* was significantly lower in saplings than in large trees (both upper and lower canopy), whereas we did not find a significant difference in the FungiQuant CT value among saplings and lower and upper canopy positions (Supplementary Table 3). In addition, correlation analysis showed that both Shannon and Simpson diversity indices had a negative correlation with *H. fraxineus* CT values (*R*^2^ = 0.2616 and *R*^2^ = 0.2762, linear regression, y = -6.9138 + 47.328 and y = -1.1924x + 38.699) (Figures 2B,C). Since CT values are inversely related to the amount of target DNA, these data indicate that an increase in *H. fraxineus* DNA amount coincided with an increase in the fungal community diversity. The CT values from the FungalQuant assay had a negative correlation with total DNA yield as measured with Qubit (*R*^2^= 0.4914, linear regression, y = -0.0345x + 31.848) (Figure 2D), indicating that an increase in total DNA yield from leaf sample coincided with an increase in fungal colonization level. No correlation was observed between FungalQuant CT values and *H. fraxineus* CT values or sequence read percent (data not shown as non-significant results were found).

### 3.3 Network structure of fungal communities associated with ash sapling and tree

**FIGURE 3:**
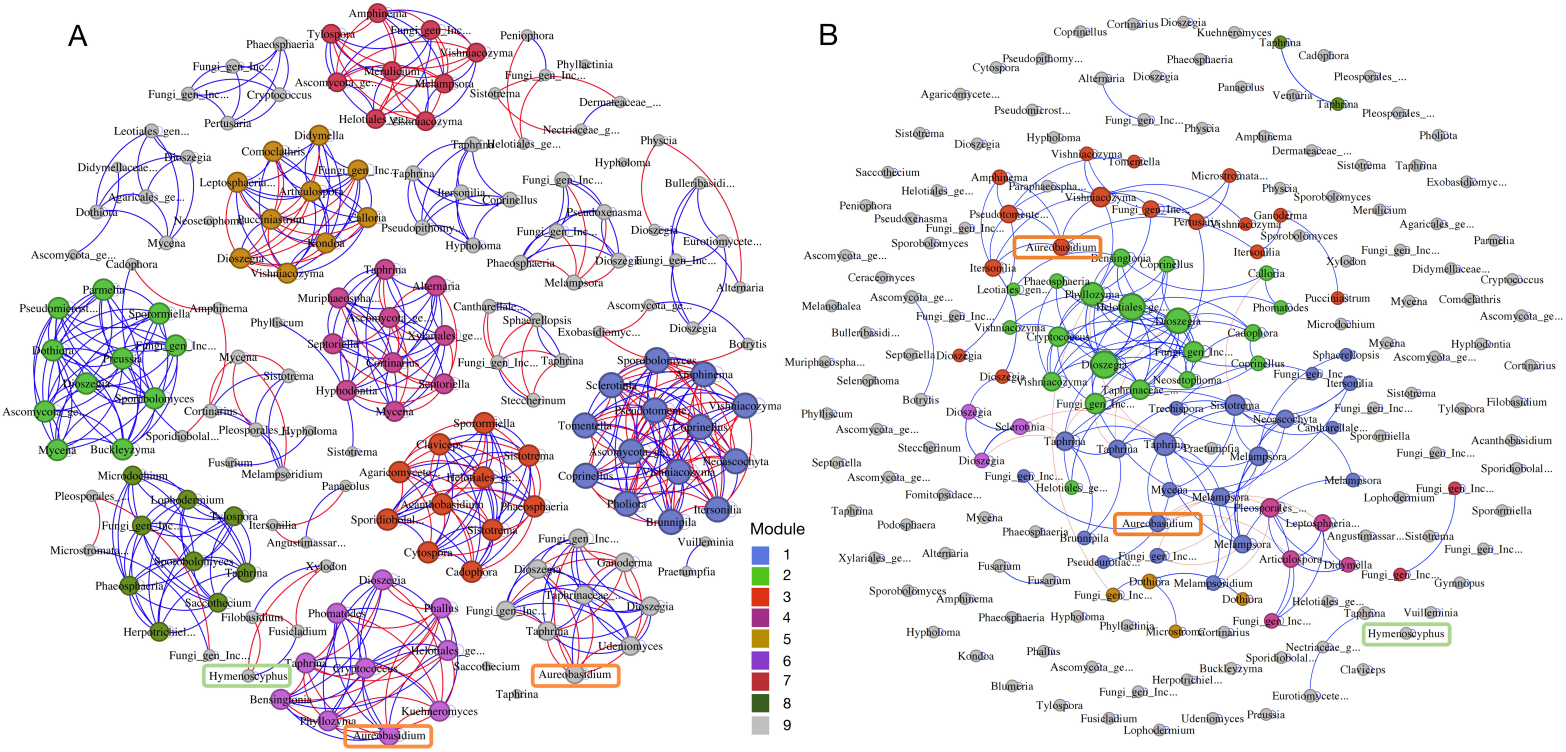
Co-occurrence network analysis of phyllosphere communities for (A) saplings and (B) mature trees. A connection stands for Spearman correlation and is statistically significant (false discovery rate-corrected *p* < 0.05). The color of the node denotes the modularity, whereas the blue and red edges denote a positive and negative correlation, respectively. The node size is proportional to the number of connections. *Hymenoscyphus* is marked by a green rounded rectangle and *Aureobasidium* is marked by an orange rounded rectangle.

Co-occurrence networks were constructed based on the OTU matrices at the genus level to characterize interactions between fungal taxa within the phyllosphere communities of saplings and mature trees (Figure 3). We found that saplings had more edges (connections) than large trees, even though saplings had fewer nodes (OTUs) than mature trees (Figures 3A,B). In addition, the fungal communities of both saplings and trees showed more positive correlations (73.26 and 95.45% respectively) than negative correlations (26.74 and 4.55% respectively) (Supplementary Table 4), indicating that most genera tended to co-occur, rather than exhibit mutual exclusion or niche separation. Furthermore, the co-occurrence networks of fungal communities in saplings had higher values for topological indices, including graph density (0.052), average degrees (8.599), and modularity (0.917) than trees (graph density = 0.019, average degree = 3.686, modularity = 0.639) (Supplementary Table 4).

The taxonomic connections within the networks were similar among saplings and large trees at class level but not at genus level. At the class level, *Agaricomycetes* had the most links with other taxa (26.88 and 27.36% of the total connections for saplings and large trees, respectively), followed by *Dothideomycetes* (22.58 and 23.58%, respectively) and *Leotiomycetes* (12.9 and 14.15%, respectively). At the genus level, *Hymenoscyphus* was positively correlated to *Fusicladium* and *Itersonilia* but negatively correlated to *Xylodon* and *Filobasidium* in the sapling fungal community network. In contrast, there was no connection between *Hymenoscyphus* and any other fungi in the investigated large ash trees.

### 3.4 Sapling leaves show higher *H. fraxineus* ascocarp production than leaves of large trees

There were no significant differences between lower and upper light canopy leaves from large trees in relation to the production of *H. fraxineus*-like ascomata; for additional comparisons, the data from lower and upper light canopy leaves concerning the presence of *H. fraxineus*-like ascocarps were pooled together for each large tree. On average, 7.5 and 1.4 *H. fraxineus* ascocarps per petiole/rachis tissue were recorded for saplings and large trees, respectively. Except for tree 4 and tree 7, the difference between saplings and large trees was statistically significant (Kruskal-Wallis and Dunn’s *post hoc*, *P* < 0.05). On average, 39 and 6% of petioles from saplings and large trees, respectively, hosted *H. fraxineus* ascocarps within the basal 5cm of each petiole—the difference was statistically significant (Kruskal-Wallis and Dunn’s *post hoc*, *P* < 0.05). While our sampling height in large trees ranged between 15 and 30 m, there were no significant differences between trees of different heights in relation to the number or location of *H. fraxineus* ascocarps (Table 1). The relationship analysis between *H. fraxineus* (CT value) and proportion of petioles with FBs at base or number of FBs per petiole showed a negative correlation, i.e., the higher the *H. fraxineus* DNA amount estimate was, the more fruiting bodies were observed (Supplementary Figure 2).

## 4 Discussion

We found clear differences in the phyllosphere fungal communities of common ash across canopy positions in a forest affected by ash dieback. Fungal diversity was highest in leaflets from understory saplings and lowest in those from the upper light canopy of mature trees (Shannon and Simpson indices). At the 2022 leaf sampling, very few saplings were alive in the studied forest, whereas the stand was healthy and supported abundant saplings in 2014, when the site was sampled for genetic population studies of ash (Tollefsrud et al., 2016). This change reflects the rapid mortality of juvenile trees compared with mature ones in diseased forests (Coker et al., 2019). It cannot be excluded that the surviving saplings include genotypes with somewhat enhanced tolerance to ash dieback, which could in turn influence their leaf mycobiome composition. Consequently, comparisons among studies should account for differences in tree size, sampling height, and stand disease history. The PhD work of Reiher (2011) and the related article by Scholtysik et al. (2012) from Germany, carried out prior to the arrival of *Hymenoscyphus fraxineus* and based on fungal culturing from surface-sterilized leaflets, are sampling-wise comparable to our study, as these authors sampled from both the light and shade canopy of tall ash trees (≥25 m) as well as from understory saplings. Similar to those studies, the diversity patterns observed here may reflect a combination of microclimatic, physiological, and methodological factors, as discussed below.

Fungal communities in the light canopy of large trees are subjected to harsh environmental conditions such as high exposure to UV radiation, oscillating nutrient and moisture availability and temperature, and nutrient limitation (Bringel and Couee, 2015). Reiher (2011) compared temperature and moisture between light and shade crown of large trees—temperature was on average 0.5°C higher and moisture 6% lower in the light canopy than in the shade crown of large ash trees, the maximum daily differences between the two canopy positions during the growing season being 4°C and 30%. They did not measure these parameters for the understory saplings, but obviously the differences are even more pronounced between the light canopy of large trees and saplings. Temperature, moisture and exposure to UV light probably influence and limit epiphytic colonization of many microorganisms and establishment of leaf infection in the light canopy, leading to a selective enrichment of specialists. It is worth noting that the dimorphic yeast *Aureobasidium pullulans*, adapted to survive on harsh oligotrophic substrates (Gostinèar et al., 2014) and probably capitalizing on nutrients leaching from plant tissues, was the most common fungus identified at genus level in the light canopy of large common ash trees with sequence read proportion of 21.7%, whereas sapling leaflets showed a sequence read proportion of 3.4% for this fungus (Supplementary Table 5). In the study of Reiher (2011), *Alternaria alternata* (Fr.) Keissl. 1912 and *A. infectoria* E.G. Simmons 1986 constituted above 60% and *A. pullulans* less than 5% of the isolates obtained in autumn from the light canopy of large common ash trees. In a study from Spain relying on fungal culturing from surface-sterilized leaves of common ash (size of sampled trees not enclosed), *Alternaria* isolates constituted approx. 40% of the isolates obtained, while no isolation of *A. pullulans* was reported (Trapiello et al., 2017). *Alternaria* species also have traits that allow persistence in stressful conditions, such as deposition of melanin on the conidial surface to confer UV tolerance (Kawamura et al., 1999). We are not aware of any metabarcoding study of leaf mycobiome of common ash where species of *Alternaria* have shown prominent sequence read proportions. Such study-specific differences in the leaf mycobiome composition may reflect local variation in the prevalence of different fungal species with affinity to common ash leaves or be due to a method bias. Culture-based isolations tend to favor fast-growing, readily sporulating taxa and underrepresent slow-growing or oligotrophic species, whereas DNA-based surveys are broader but can be affected by primer bias and DNA-extraction efficiency. For example, *Alternaria alternata* strains (Ahmad et al., 2024) exhibit several-fold higher mycelial growth rates on laboratory media than strains of *Aureobasidium pullulans* (Iqbal et al., 2023). Beyond methodological effects, phyllosphere communities also vary over time: assemblages shift with season and with leaf ontogeny, in addition to changes associated with *H. fraxineus* presence and environmental conditions. These factors likely contribute to the differences among studies.

Fungal species-specific differences in the life cycle may also contribute to the observed decline in fungal community diversity and differences in species composition along with sampling height. The proximity of foliage to the fungal species-rich litter (Collado et al., 2007) and soil (Buée et al., 2009) may be one determinant, especially in relation to fungi with fructification only during the saprophytic phase in leaf litter, such as *H. fraxineus*. Chandelier et al. (2014) recorded up to 100-fold lower spore concentration of *H. fraxineus* at a height of 3 m above ground in comparison to a height of 0.5 m above ground in common ash forests. In consistency, the now recorded sequence read proportions of *H. fraxineus* were 10.5, 3.6, and 1.7% for leaflets from saplings and the lower and upper light canopy of large common ash trees, respectively. Overwintering in buds could provide an additional source of inoculum for leaf-associated fungi, but the extent to which these fungi could overwinter in buds is poorly known for common ash. The PhD study of Chen (2012) from New Zealand, where common ash is introduced and ash dieback absent, showed the presence of *A. pullulans*, basidiomycetous yeasts, and several filamentous ascomycetes in buds of this tree species. It is noteworthy that Chen (2012) detected the presence of *A. pullulans* by DNA sequencing of cloned ITS PCR products but not by fungal isolation. In our study, *A. pullulans* remained very common in the upper canopy leaves, consistent with niche partitioning and priority effect from early colonization (potentially including bud sources). However, it should be kept in mind that metabarcoding data are relative, a high *A. pullulans* relative abundance does not necessarily correspond to a high biomass of this species in the tissue (Gloor et al., 2017; Lofgren et al., 2019).

The observed predominance of *Ascomycota* over *Basidiomycota* in the foliage of saplings and mature trees, with sequence read proportions of 62.3 vs. 33.1% and 85.1 vs. 11.5%, respectively, is consistent with the findings of previous leaf mycobiome studies of common ash. In the study of Reiher (2011) mentioned above, *Ascomycetes* constituted more than 90% of the isolates obtained from saplings and light shadow canopy of large common ash trees, the proportions of *Basidiomycetes* being well below 10%. Becker et al. (2020), in a cultured-based approach, reported isolation frequencies of 72 and 28% for *Ascomycetes* and *Basidiomycetes*, respectively, from leaves taken from “the middle part of crowns” of healthy and diseased common ash trees with age in the range between 60 and 80 years. In the metabarcoding part of their study, Becker et al. (2020) obtained sequence read proportions of 61 and 38% for *Ascomycetes* and *Basidiomycetes*, respectively. Concerning metabarcoding of mycobiome associated with leaves of common ash saplings or young trees (around 30 years old), Agan et al. (2021) and Agostinelli et al. (2021) reported *Ascomycetes* and *Basidiomycetes* sequence read proportions of 62 vs. 37% and 59 vs. 39%, respectively. The differences among studies likely reflect methodological and ecological factors.

The observed relatively high proportion of *Basidiomycetes* in saplings (33.1%) in comparison to the upper canopy of large common ash trees (11.5%) mirrors the sequence read proportions of *H. fraxineus*, which were 10.5% for saplings and 3.6% for the lower light canopy and 1.7% for the upper light canopy of large trees. The necrotic lesions caused by *H. fraxineus* (Lygis et al., 2017; Schwanda and Kirisits, 2016) may create moist microsites that, similar to other decaying plant tissues, could favor the proliferation of basidiomycetous yeasts (Lindow and Brandl, 2003; Gouka et al., 2022). This pathogen-mediated disturbance may therefore coincide with a higher Basidiomycota fraction in saplings. A similar pattern of co-variation between *H. fraxineus* and *Basidiomycetes* was seen in the study by Griffiths et al. (2019), who used metabarcoding to examine mycobiome and bacteriome in leaves of saplings and mature (sampling height not enclosed) common ash in the UK. In their study, the read proportions of *Basidiomycetes* were 43% in leaves with no *H. fraxineus* DNA and 52% in leaves with a high level of *H. fraxineus* DNA. Another similarity to our study was that higher *H. fraxineus* DNA levels were positively correlated with fungal community diversity and that with high *H. fraxineus* DNA level the leaf microbial networks were characterized by stronger associations between fewer members than those associated with low *H. fraxineus* DNA levels. Griffiths et al. (2019) proposed that *H. fraxineus* disrupts stable endophyte communities after a particular infection threshold is reached, this leading to proliferation of opportunistic microbes. We find this a realistic scenario but offer some additional reflections. In the present work, leaf sampling was carried out in mid-September, and by this time, most of the leaves of saplings had already been shed, and the remaining ones that were sampled showed very low retention upon removal from the shoot, whereas the leaves of large trees were very firmly attached to the shoots. This would imply that by the time of sampling, leaf senescence processes had advanced further in the saplings in comparison to the large trees. Interestingly, the FungiQuant real-time PCR employed to estimate leaf-associated fungal biomass in leaflets of saplings and large trees indicated that they hosted comparable levels of fungal propagules. In leaf tissues, *H. fraxineus* mycelia shows a high affinity to starch-rich cells that are crucial for a plant’s energy management and survival (Nielsen et al., 2022). It seems reasonable to propose that the high load of *H. fraxineus* propagules in sapling leaves may have contributed to a premature breaching of a critical threshold in leaf microbial carrying capacity and triggered the onset of leaf senescence.

Somewhat similar to Griffiths et al. (2019), it is tempting to propose that the higher modularity of fungal network in sapling leaflets in comparison to those of large trees could be related to a more advanced senescence stage of the former, as one might expect more pronounced species interactions during the microbial race to secure nutrients and territory in weakened tissues. These interactions may include positive metabolic relationships, such as cross-feeding. Microbial cross-feeding has been well-documented in bacterial communities (Zelezniak et al., 2015) and fungus-bacteria consortia (Velez et al., 2018), where one species produces metabolites or compounds that another species needs for its growth, while the recipient species produces substances that the first species requires. Although direct evidence of fungus–fungus cross-feeding is scarce, the presence of modules with positive correlations between filamentous basidiomycetes and basidiomycetous yeasts in sapling leaves could represent cross-feeding relationships—for example, yeasts feeding on filamentous basidiomycete-generated degradation products from structural carbohydrates present in leaf tissues, while the latter utilize yeast-excreted metabolites, such as alcohols or organic acids as additional energy sources.

Negative correlations between a pathogen such as *H. fraxineus* and other fungal species could be used for understanding biological control and disease suppression (Poudel et al., 2016) in network analysis. In the present study, the co-occurrence analysis indicated that saplings with a high level of *H. fraxineus* DNA had a highly connected and complicated network, while the large tree with a lower level of *H. fraxineus* DNA had a minimally connected network. Specifically, we found a negative correlation between *H. fraxineus* and two fungal genera, *Filobasidium* and *Xylodon*. Several prior metabarcoding studies on ash leaf mycobiome have also considered whether indications of any antagonistic effect could be derived from a negative frequency relationship between *H. fraxineus* and other leaf inhabitants, but with inconsistent results. In a study in Switzerland, Schlegel et al. (2018) found a weak negative association between *H. fraxineus* and an unidentified species of *Setophoma* sp., while Agan et al. (2021) found a negative correlation between *H. fraxineus* and three fungal species (*Taphrina padi*, *Vishniacozyma laurentii*, *Rhodosporidiobolus colostri* in leaflets of common ash from Estonia, while leaflets from Norway also considered in that study showed no fungi with negative correlation with *H. fraxineus*. The inconsistencies between studies may relate to differences in sampling time, sample processing and methodological biases, but may also reflect variation in local fungal species composition or relate to stochasticity in microbial species arrival order on individual leaves and associated priority effects (niche preemption and niche modification; Fukami, 2015), but also the scale of sampling. Since both *A. pullulans* (McGrath and Andrews, 2006) and *H. fraxineus* (Nielsen et al., 2022) have specific preference for the leaf vein system, a question of the nature of their relation arises. *Aureobasidium pullulans* can display antagonistic activity toward several phytopathogenic fungi (Schena et al., 2002; Bozoudi and Tsaltas, 2018). In the study of Schlegel et al. (2016) exudates from *A. pullulans* did not have any impact on *H. fraxineus* ascospore germination. However, it remains possible that *A. pullulans* could inhibit mycelial growth of the pathogen. No relation between *A. pullulans* and *H. fraxineus* was observed in the here presented fungal co-occurrence analysis. Comparison of mycobiome associated epiphytically or endophytically with ash leaflet nerves between healthy and symptomatic trees might be a more valid orientation basis in future studies.

We found that leaves of both saplings and large common ash trees support the production of *H. fraxineus*-like fruiting bodies. However, sapling leaf petioles had a significantly higher number of *H. fraxineus*-like fruiting bodies than large tree leaf petioles after 1 year of incubation on the forest floor. This is coherent with the vertical decline in *H. fraxineus* ascospore level in diseased forests (Chandelier et al., 2014), and the observed higher sequence reads of *H. fraxineus* and lower CT-values by the *H. fraxineus* DNA-specific real-time PCR in sapling leaves in comparison to leaves from large common ash trees. Thus, it seems obvious that in the now examined multilayered ash forest, the saplings were exposed to a much higher density of pathogen ascospores than large trees with the light canopy at 20–30 m. Despite the variation in defoliation levels among the considered large trees, we did not find any statistically significant patterns in the number (or location in relation to petiole base) of *H. faxineus*-like fruiting bodies produced by their leaves after overwintering, but there was a pattern that trees with high defoliation rate tended to have a higher proportion of pathogen ascomata close to the petiole base than healthier trees. This observation is worth a further investigation as mycelial growth past petiole base prior to autumn leaf shed is considered a major path for shoot infection by *H. fraxineus* (Haňáčková et al., 2017). The study of Nielsen et al. (2017) suggested that susceptibility to leaf infection and to shoot dieback are regulated by different mechanisms as ash species with no shoot dieback can support abundant formation of *H. fraxineus* ascomata, but the underlying mechanisms remain poorly understood.

In a recent study, Marçais et al. (2023) showed that healthy saplings of common ash could act as cryptic disease reservoirs. Our results reinforce that infection pressure in ash stands is not confined to visibly diseased trees. Both saplings and mature ash, regardless of health status, contribute to the production of *Hymenoscyphus fraxineus* fruiting bodies; our data suggest that foliage biomass across canopy layers might be a key determinant of local infection pressure. This provides direct evidence that in multilayered ash forests, dominant healthy trees can sustain intense local inoculum pressure.

Juvenile development and survival are critical for the long-term persistence of ash. In forest stands affected by ash dieback, the next generation of ash can become genetically enriched for alleles associated with disease tolerance, reflecting pathogen-driven selection against progeny of weaker trees (Metheringham et al., 2025). However, seedlings and saplings are particularly vulnerable due to their small canopy size and greater exposure to inoculum from the forest floor. Even if parental trees are partially tolerant, their offspring face much higher pathogen loads and more conducive infection conditions near the forest floor. Thus, the potential for offspring to reach sapling, pole and large tree stages could be constrained by intense exposure to *H. fraxineus* ascospores in forests with high ash foliage biomass, despite any selection pressure by the pathogen.

Our study and that of Marçais et al. (2023) raise important questions about the future of multilayered ash stands under ash dieback. Current guidance promotes a risk-based approach—with focus on retaining structurally sound or tolerant trees while only removing hazardous or severely diseased individuals—to maintain genetic diversity and facilitate natural selection for tolerance (Enderle et al., 2017a,b). Balancing the retention of potential “healthy carriers” with the need to sustain a viable gene pool probably requires nuanced, site-specific strategies that integrate both genetic and epidemiological dimensions while keeping infection pressure at a sustainable level. Future research should examine how inoculum dynamics across canopy and understory layers influence ash regeneration and the development of seedlings through successive growth stages.

## 5 Conclusion

In summary, the study of the common ash leaf fungal community indicates that fungal composition and diversity in ash saplings and large mature trees differ from each other significantly, and that saplings have the most diverse fungal communities in comparison to the lower and upper light canopy of large trees. Moreover, fungal alpha diversity is associated with the level of *H. fraxineus*. Saplings with higher levels of *H. fraxineus* exhibit more complex fungal co-occurrence networks, characterized by higher modularity and more connections. In contrast, large ash trees with lower levels of *H. fraxineus* show more stable networks, with lower modularity and fewer connections. Furthermore, the number of *H. fraxineus* ascocarps produced on petiole/rachis tissues in saplings differs significantly from most of the large trees. It is notable that leaves from large trees with no defoliation also produced *H. fraxineus* ascocarps. This supports previous results that healthy carriers of *H. fraxineus* exist and might play an important role in ash dieback epidemiology. Future studies on infection pressure dynamics across forest strata may prove important for a better understanding of successful ash regeneration and survival through the critical seedling and sapling phases.

## Data Availability

All sequence data is available at NCBI under PRJNA1177464.

## Footnotes

1
https://jgi.doe.gov/data-and-tools/software-tools/bbtools
