# Supplementary material for: Leaf mycobiome and the success of Hymenoscyphus fraxineus in completing its life cycle depend on the canopy position of common ash
Source: Front Microbiol. 2025 Dec 9;16:1696858. doi: 10.3389/fmicb.2025.1696858 (PMC12722789; doi:10.3389/fmicb.2025.1696858)
Supplement: Supplementary file 1 [file Data_Sheet_1.docx]

Supplementary Material

# Supplementary Figures and Tables

## Supplementary Figures


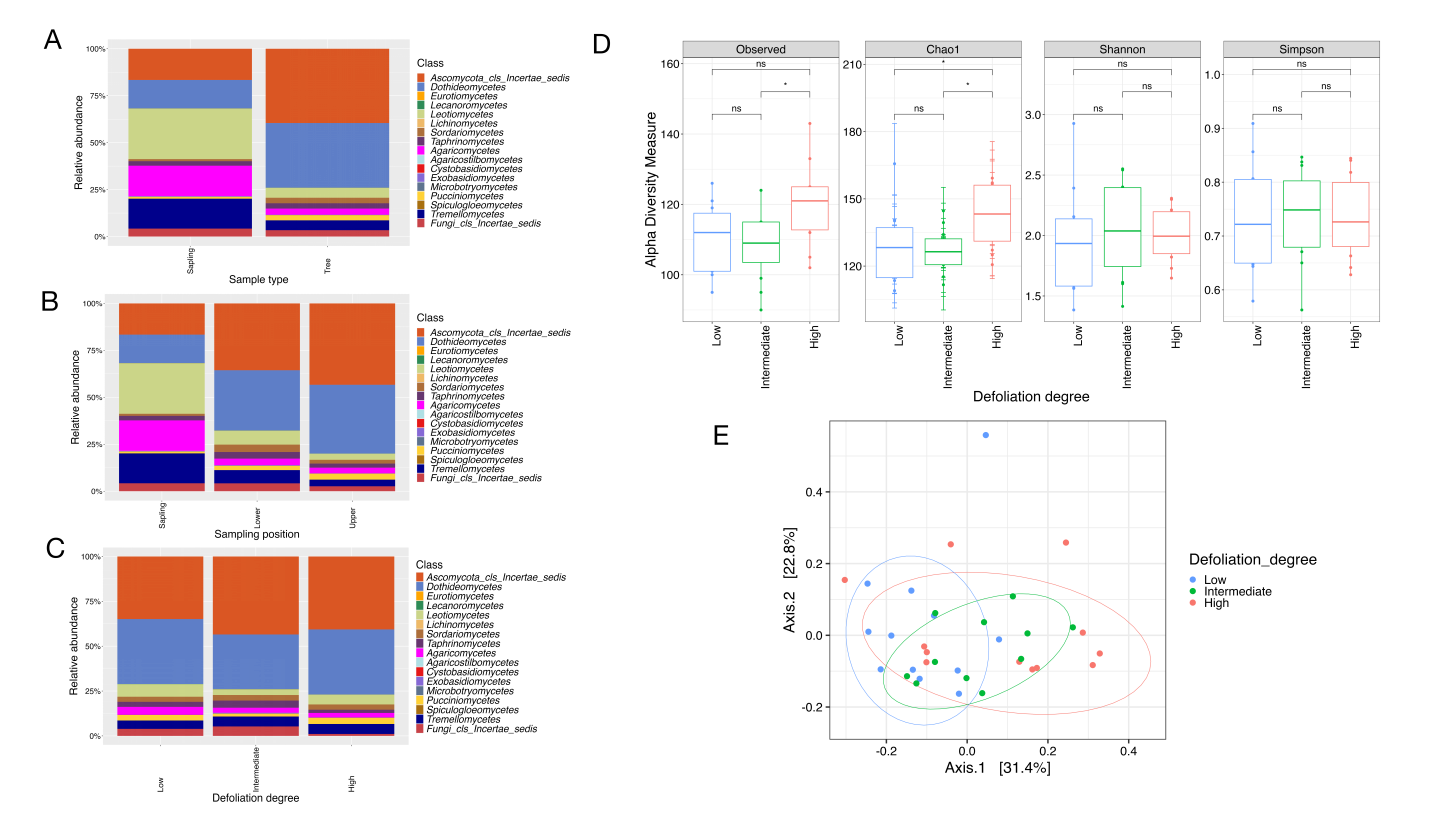


**Figure S1.** (A)(B)(C) Relative abundance of the most common fungal taxa at class level in ash leaflet samples (based on sampling positions and defoliation degree). The group “other” includes all the least prevalent fungal genera. (D) Fungal community diversity, including richness (observed OTUs, Chao1), diversity (Shannon and Simpson index). (E) Principal coordinates analysis (PCoA) of phyllosphere mycobiome based on Bray-Curtis dissimilarity. Plot illustrating distances between communities in all individual samples for fungi. The ellipses represent 95% confidence intervals for different defoliation degree

**
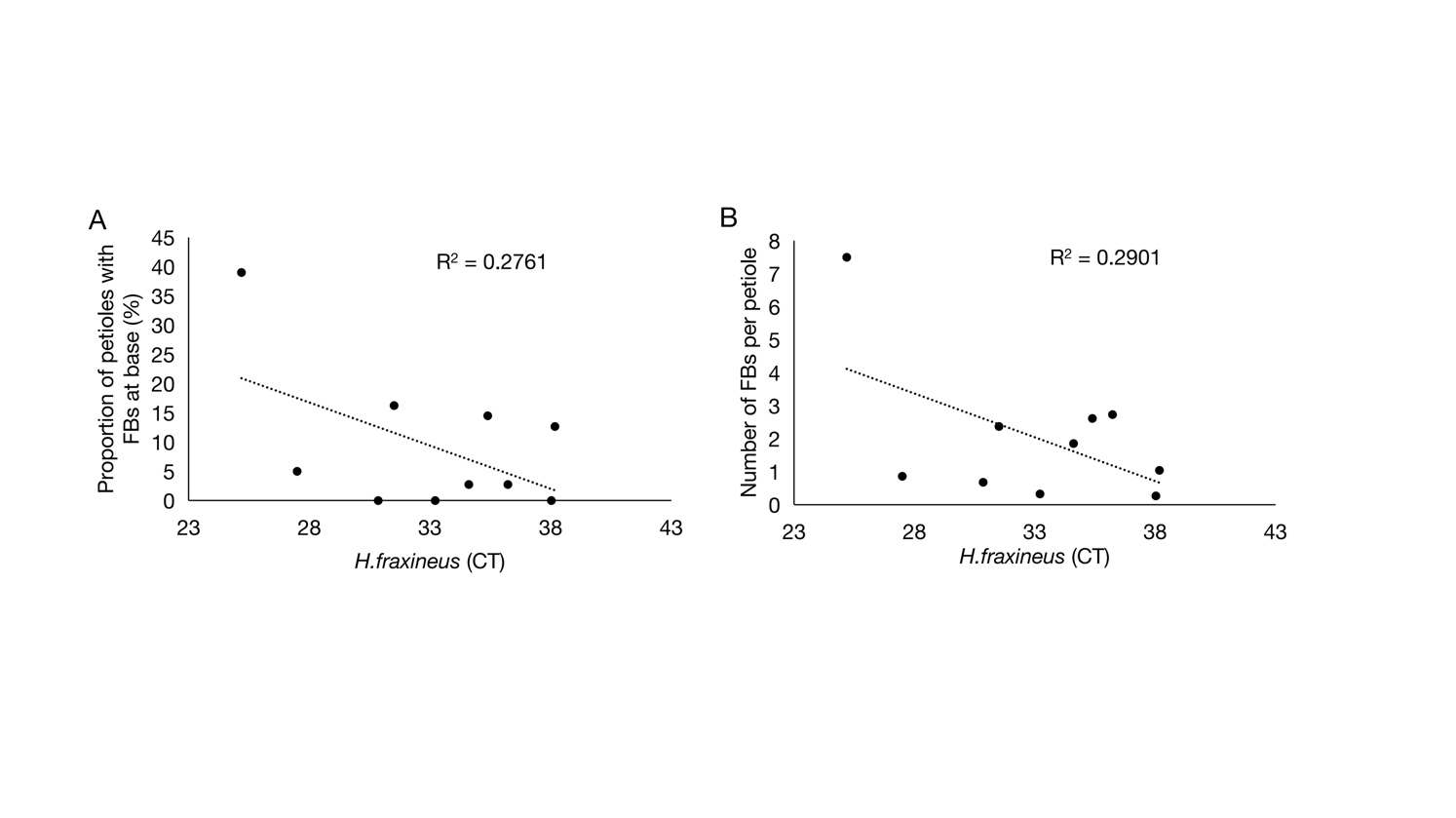
**

Figure S2. Relationship between *H. fraxineus* (CT value) and (A) proportional of petioles with *H. fraxineus*-like fruiting bodies (FBs) at base or (B) Number of FBs per petiole.

## Supplementary tables

Table S2. Permutational multivariate analysis of variance (PERMANOVA) table showing the factors explaining fungal assemblage structure in large tree.

|  | Df | SumOfSqs | R^2^ | F | Pr(>F) |
| --- | --- | --- | --- | --- | --- |
| Sampling position | 1 | 0.2696 | 0.0858 | 3.3322 | 0.002 |
| Defoliation degree | 2 | 0.4207 | 0.1339 | 2.5999 | 0.009 |
| Sampling position:Defoliation degree | 2 | 0.1055 | 0.0336 | 0.6519 | 0.804 |
| Residual | 29 | 2.3465 | 0.7467 |  |  |
| Total | 34 | 3.1423 | 1 |  |  |

Table S3. The comparison of *Hymenoscyphus fraxineus* and FungiQuant in different canopy position.

| Canopy position | *H. fraxineus* (%) | *H. fraxineus* (CT) | FunqiQuant (CT) |
| --- | --- | --- | --- |
| Tree-upper | 1.74a | 34.45a | 30.79a |
| Tree-lower | 3.55ab | 33.16a | 30.42a |
| Sapling | 10.5b | 25.19b | 30.73a |

Note: the small letters are showing the significance among canopy positions.

Table S4. Co-occurence network topological properties of phyllosphere fungal communities in saplings and large trees.

| Network indicator | OTU matrix | |
| --- | --- | --- |
|  | Sapling | Tree |
| Total Nodes | 167 | 191 |
| Total edges | 718 | 352 |
| Average degree | 8.599 | 3.686 |
| Positive correlation edge propotion (%) | 73.26 | 95.45 |
| Negative correlation edge propotion (%) | 26.74 | 4.55 |
| Graph density | 0.052 | 0.019 |
| Number of modlues | 33 | 122 |
| Average clustering coefficient | 0.988 | 0.645 |
| Modularity | 0.917 | 0.639 |

Table S5. Top 20 most common OTUs in three forest canopy positions at genus level.

| OTUs | Genus | Sapling  reads | Lower canopy  reads | Upper canopy reads |
| --- | --- | --- | --- | --- |
| OTU1 | *Ascomycota_gen_Incertae_sedis* | 9514 | 86929 | 111891 |
| OTU2 | *Aureobasidium* | 1953 | 39851 | 69306 |
| OTU3 | *Fusicladium* | 709 | 19639 | 11691 |
| OTU4 | *Hymenoscyphus* | 6077 | 8683 | 4514 |
| OTU5 | *Fungi_gen_Incertae_sedis* | 2017 | 7204 | 4614 |
| OTU6 | *Nectriaceae_gen_Incertae_sedis* | 618 | 8811 | 5104 |
| OTU7 | *Melampsora* | 297 | 2989 | 4518 |
| OTU8 | *Praetumpfia* | 98 | 2809 | 10410 |
| OTU9 | *Dioszegia* | 1527 | 3877 | 1833 |
| OTU10 | *Melampsora* | 209 | 2312 | 3386 |
| OTU11 | *Didymella* | 3176 | 7713 | 178 |
| OTU12 | *Sclerotinia* | 3556 | 6522 | 2883 |
| OTU13 | *Ganoderma* | 2627 | 4710 | 3146 |
| OTU14 | *Itersonilia* | 1310 | 4550 | 2945 |
| OTU15 | *Taphrina* | 37 | 1438 | 2874 |
| OTU16 | *Leptosphaeriaceae_gen_Incertae_sedis* | 2091 | 6191 | 607 |
| OTU17 | *Taphrina* | 258 | 2938 | 728 |
| OTU18 | *Phyllactinia* | 5382 | 1346 | 414 |
| OTU19 | *Taphrina* | 1034 | 3877 | 1291 |
| OTU20 | *Cryptococcus* | 134 | 2473 | 1281 |
